# Reversible Acute Kidney Injury and Rhabdomyolysis Due to Severe Autoimmune Hypothyroidism in an Adolescent Using Creatine Supplements

**DOI:** 10.7759/cureus.100875

**Published:** 2026-01-05

**Authors:** Tuqa A Abdulsalam, Razan Ismail

**Affiliations:** 1 General Pediatrics, Al Jalila Children's Speciality Hospital, Dubai, ARE; 2 Emergency Department, Al Jalila Children's Speciality Hospital, Dubai, ARE

**Keywords:** acute kidney injury, autoimmune hypothyroidism, creatine supplementation, dehydration, elevated creatine kinase, hypothyroidism, hypothyroid myopathy, non-traumatic rhabdomyolysis, rhabdomyolysis

## Abstract

Profound hypothyroidism is a rare but reversible etiology of acute renal failure (ARF) associated with rhabdomyolysis, and it occurs mainly in children or adolescents. We describe a 16-year-old previously healthy male who presented with progressive fatigability, hypersomnia, myalgia, cold intolerance, and dark-colored urine, along with raised serum creatinine levels, in the context of extreme dieting, dehydration, and creatine intake. Laboratory examination showed severe autoimmune hypothyroidism with a thyroid-stimulating hormone (TSH) level of >100 μIU/mL, significantly low free T4 and T3 concentrations, positive results for anti-thyroid peroxidase and anti-thyroglobulin antibodies, and elevated creatine kinase indicative of rhabdomyolysis in the setting of concurrent acute kidney injury (AKI). The patient was treated with intravenous hydration, levothyroxine replacement, and vitamin D supplementation and instructed not to use creatine. His clinical and biochemical condition improved rapidly, accompanied by gradual recovery of the renal function and disappearance of the musculature symptoms. Outpatient endocrine follow-up revealed ongoing recovery. Our case presents the role of hypothyroidism in such unexplained AKI with raised creatine kinase, especially in adolescents using performance-enhancing supplements and involved in restrictive dietary practices.

## Introduction

Hypothyroidism is a common endocrine disorder and results from an inadequate synthesis of thyroid hormone, leading to the disruption of systemic physiological functions. Though cardiovascular, metabolic, and neuromuscular symptoms are commonly associated with hypothyroidism, the renal effects of this disease often remain invisible clinically, especially in pediatrics and adolescence. Renal hemodynamics, glomerular filtration rate (GFR), and tubular function are profoundly affected by thyroxine through its effect on cardiac output, renal blood flow, systemic vascular resistance, and renin-angiotensin-aldosterone system (RAAS) activity [1,2]. For instance, the renoprotection provided by hypothyroidism against GFR and serum creatinine is lost when patients develop hypothyroid-induced organ failure, such as renal medullary aplasia, or receive treatment with thyroid hormone replacement [1-3].

Several clinical trials have confirmed an association of hypothyroidism and a functional, reversible elevation in serum creatinine without structural kidney disease. Kreisman and Hennessey, showing the fact that serum creatinine levels are definitely elevated in hypothyroid patients and also come down to normal levels once a state of euthyroidism is achieved, presented solid evidence for a cause-and-effect association between hypothyroidism and the presence of renal failure [2]. During this reversible reduction in renal function, in the context of severe or prolonged hypothyroidism, there is potential for progression to acute kidney injury (AKI), particularly if there are contributing insults such as dehydration, rhabdomyolysis, infection, or nephrotoxin exposure [4,5].

Muscle involvement in the hypothyroid state includes muscle metabolic status as another well-established but heterogeneous feature. Patients often present with complaints of cramps, aches, stiffness, or delayed reflexes (as probably secondary to the injury of muscle cell membranes), as well as proximal limb muscle weakness and increased serum creatine kinase (CK) biological activity [6]. Furthermore, hypothyroidism may rarely induce rhabdomyolysis, a life-threatening syndrome caused by myocyte necrosis and massive CK and myoglobin release into circulation with pigment-induced tubular injury and AKI [6]. Unlike the usual causes of rhabdomyolysis, such as exertion or toxins, hypothyroid-induced rhabdomyolysis can occur without any traumatic event or significant exertion, which may lead to a delayed diagnosis [2,5].

The synergistic actions are based on the renal perfusion by hypothyroidism and tubular toxicity via rhabdomyolysis, which is a pathway for AKI induction [4]. We found that the partial and complete resolution of this condition through hormone replacement therapy and aggressive hydration has been described in several case series and reviews, emphasizing the importance of early recognition for its reversibility [5].

Over the past few years, creatine supplements have become more and more widely accepted among adolescents and young athletes for their effect on promoting muscle mass growth [5]. Oral supplementation with creatine enhances the serum level of creatinine by a nonenzymatic transformation, which can mask renal failure because creatinine is used as an indicator for diagnosing. Moreover, creatine itself may become the target for rhabdomyolysis and AKI during dehydration, starvation, or rigorous exercise [6]. Life-threatening dietary supplement use is a greater concern when teenagers outside of competitive athletics are taking them unsupervised because they have limited physiological reserves and concomitant disordered eating.

The presence of severe autoimmune hypothyroidism, rhabdomyolysis, and dehydration, as well as creatine being a concomitant etiology for AKI in this case, is very rare in the pediatric literature. In most of the reported cases, they are either in adult patients or with isolated hypothyroidism-induced renal impairment alone without other metabolic disturbances [6].

We present this case to demonstrate the intricate, multifactorial pathophysiology of AKI in severe hypothyroidism, bring attention to the potential dangers of self-supplementation with creatine among adolescents and emphasize the critical addition of screening for thyroid function to one's diagnostic workup for an unexplained AKI with elevated creatine kinase.

## Case presentation

A previously healthy 16-year-old adolescent attended the emergency department due to one month of fatigue, excessive daytime sleepiness, loss of appetite, poor school performance, and generalized body pains, all of which worsened over the last month, particularly in the past two weeks. He had symptoms consistent with hypothermia, cold intolerance, bilateral calf pain (left more than the right), dark and concentrated urine, and difficulty rising from the squatting position. The patient did not have fever, rash, vomiting, diarrhea, dysuria, or hematuria, and there was no night sweat or unintentional weight loss. However, the patient admitted to a 6-kg weight loss in the past month that was intentional in nature through strict dieting and frequent meal replacement with chewing gum. The patient also reported on-and-off use of creatine powder (a gym-based supplement) for approximately one month daily, frequently dry scooping without water, and noted suboptimal regular hydration. He had been going to the gym occasionally, but discontinued as fatigue increased.

Family history included autoimmune thyroid disease and goiter in the mother and type 1 diabetes mellitus in the father. At admission, he was overweight (80 kg) but afebrile and hemodynamically stable. On exam, the patient was noted to have neck fullness without a palpable goiter, mild bilateral calf tenderness, and mild weakness rising from squatting with partial assistance. There was no peripheral edema, skin rash, or focal neurology deficit; cardiopulmonary and abdominal examinations were otherwise unremarkable.

Initial laboratory studies showed profound hypothyroidism, along with markedly positive anti-thyroid peroxidase (TPO) antibody and positive anti-thyroglobulin antibody, which confirmed autoimmune thyroiditis. A test of his kidney function indicated that his serum creatinine level was higher than normal (estimated GFR of approximately 50 mL/min/1.73 m², consistent with Kidney Disease: Improving Global Outcomes (KDIGO) stage G3a). Creatine phosphokinase was elevated in favor of myositis/rhabdomyolysis. A urinalysis revealed dark yellow urine with a specific gravity of 1.029, protein of 1+, and ketone of 1+ without hematuria. His vitamin D was 11.3 ng/mL, and HbA1c was 5.7%, indicating the prediabetes stage. Electrolytes, phosphate, uric acid, and parathyroid hormone in serum were within the normal range (Table 1). 

**Table 1:** Laboratory findings at presentation demonstrating severe autoimmune hypothyroidism complicated by rhabdomyolysis and acute kidney injury. TSH: thyroid-stimulating hormone; FT4: free thyroxine; FT3: free triiodothyronine; anti-TPO: anti-thyroid peroxidase antibody; anti-TG: anti-thyroglobulin antibody; CK: creatine kinase; AKI: acute kidney injury; AST: aspartate aminotransferase; ALT: alanine aminotransferase.

Parameter	Result	Reference range
TSH	>100 μIU/mL	0.5–4.5 μIU/mL
Free T4	1.0 pmol/L	10–22 pmol/L
Free T3	1.2 pmol/L	3.1–6.8 pmol/L
Anti-TPO antibody	242 IU/mL	<35 IU/mL
Anti-TG antibody	572 IU/mL	<40 IU/mL
Serum creatinine	1.35 mg/dL	0.6–1.2 mg/dL
Creatine kinase (CK)	1,560 U/L	30–200 U/L
AST	79 U/L	10–40 U/L
ALT	32 U/L	7–56 U/L
Urine specific gravity	1.029	1.005–1.030

**Figure 1:**
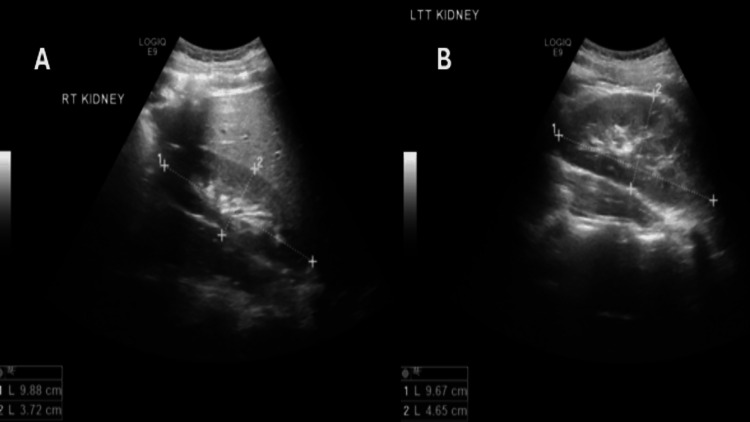
Renal ultrasound. (A) Right kidney; (B) left kidney: showing bilaterally normal-sized kidneys with mildly increased cortical echogenicity, preserved corticomedullary differentiation, and no hydronephrosis, supporting a non-obstructive, potentially reversible cause of acute kidney injury.

The patient was admitted as a case of severe autoimmune hypothyroidism, acute renal failure, and hypothyroid myopathy associated with rhabdomyolysis. He was kept on intravenous hydration and levothyroxine (initially 100 μg/day) and vitamin D (50,000 IU once a week). Consultation with nephrology and endocrinology occurred promptly. Renal ultrasound showed normal-sized kidneys with slightly increased cortical echogenicity and corticomedullary differentiation, and no hydronephrosis (Figure 1).

**Figure 2:**
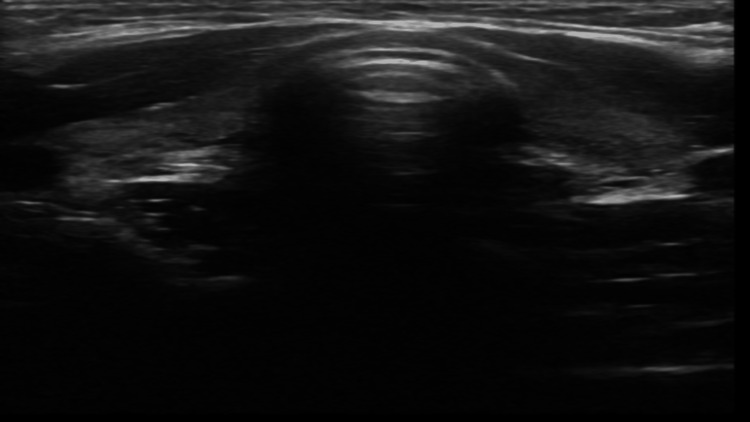
Thyroid ultrasound demonstrating bilaterally small, heterogeneous thyroid glands without focal nodules or increased vascularity, findings consistent with chronic autoimmune thyroiditis.

The ultrasound of the thyroid gland showed small glands without focal lesions or vascularization (Figure 2).

**Figure 3:**
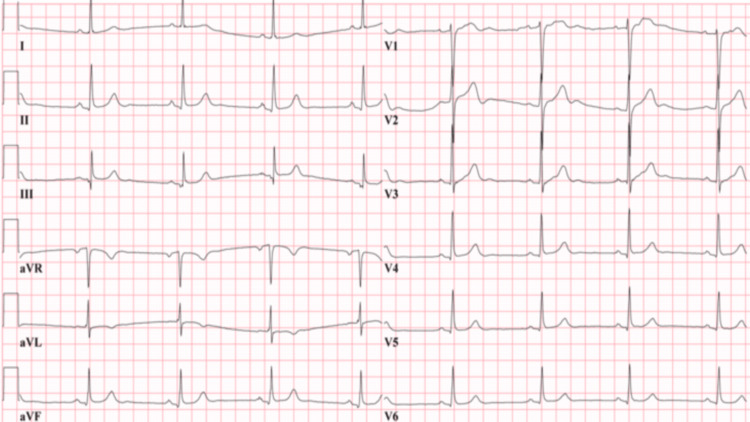
ECG showed sinus bradycardia, normal QTc interval.

There was no pericardial effusion detected by echocardiography, and the ECG showed sinus bradycardia without QT prolongation (Figure 3). 

At discharge, his clinical condition had improved gradually with restored appetite and energy and resolution of abdominal pain and myalgia. Serum creatinine dropped to 1.26 g/dL and then to 1.19 g/dL prior to discharge. He was discharged after 48 hours, discouraged the use of creatine, ensured there were no signs of dehydration, and scheduled an endocrine follow-up.

At two weeks post-discharge in the endocrinology outpatient clinic, his symptoms had substantially resolved; fatigue, myalgias, and symptoms of improved appetite, as well as resuming regular school activities. He denied experiencing additional cold intolerance, dark urine, or exercise intolerance, and he reported that he had stopped taking all creatine while ensuring adequate daily fluid intake. Serial laboratory results indicated a trend of gradual recovery in serum creatinine and an early biochemical response to levothyroxine therapy. Levothyroxine was continued, and lifestyle counseling was also re-emphasized.

## Discussion

This case illustrates an uncommon, yet clinically important, presentation of severe autoimmune hypothyroidism complicated by rhabdomyolysis and acute kidney injury (AKI) in an adolescent patient with underlying risk factors such as stringently limiting caloric intake, dehydration, and creatine use. Hypothyroidism, a prevalent endocrine disorder, has not garnered much attention as a contributing factor to clinically significant AKI in children and adolescents. Thyroid hormones are essential in renal physiology by influencing cardiac output, renal blood flow (RBF), systemic vascular resistance, and the renin-angiotensin-aldosterone system (RAAS); therefore, thyroid hormone insufficiency may reduce glomerular filtration rate (GFR) with subsequent increased serum creatinine even in patients without inherent kidney pathology [1,2].

Several studies have established that renal dysfunction in hypothyroidism is reversible. Kreisman and Hennessey have shown that serum creatinine levels remain elevated during hypothyroidism and return to normal levels with the restoration of euthyroidism. Thus, studies support a direct physiological correlation between thyroid hormone deficiency and renal function [2]. Similarly, Poortmans et al. demonstrated that untreated hypothyroidism may lead to a biochemical profile that mimics primary renal disease but reverses with levothyroxine therapy [4]. In our patient, serum creatinine decreased after intravenous hydration and the start of thyroid hormone therapy shortly afterwards, which is also suggestive of a primarily functional/hemodynamic mechanism underlying the AKI.

Muscle-related symptoms are a common but inconsistent feature of myxedema, including mild myalgia and weakness through severe hypothyroid myopathy and rhabdomyolysis. Hypothyroidism adversely affects the mitochondrial oxidative metabolism, glycogenolysis, and muscle fiber contractility and hence causes injury to the myocytes with an increase in creatine kinase (CK) [6]. In the minority of cases, hypothyroidism may trigger frank rhabdomyolysis with intense CK elevation and subsequent release of myoglobin leading to pigment-induced tubular damage and AKI [6]. The findings of elevated CK levels, calf pain, and dark urine indicated hypothyroid-induced rhabdomyolysis, which may have made a considerable contribution to the AKI via myoglobin-mediated renal tubular toxicity in our patient.

The AKI in this patient likely had a multifactorial etiology, with hypothyroidism-related decrease in renal perfusion and GFR, rhabdomyolysis-induced tubular injury, and dehydration related to restrictive dieting and poor oral intake of fluids, as well as the role of creatine supplementation [1-4]. This combination creates a “perfect storm” that fuels renal injury, while a kidney disease state is not necessary at all. Rapid resolution of the renal dysfunction when hydration and levothyroxine therapy were completed corroborates this synergistic and reversible pathophysiology, rather than irreversible intrinsic renal injury.

Adolescents and young athletes frequently use creatine supplementation for the purpose of increasing muscle mass. Creatine is non-enzymatically converted to creatinine, which can falsely increase SCr without a corresponding reduction in GFR [6]. Although creatine is regarded as safe for well-hydrated normal individuals, administration under conditions of dehydration, caloric deprivation, or the presence of any metabolic disease increases the risk for both AKI and rhabdomyolysis [5]. Cases of AKI and rhabdomyolysis have been reported secondary to creatine consumption in dehydrated individuals, especially when associated with strenuous exercise or food deprivation [6]. In our patient, the factor of dry scooping creatine without sufficient fluid intake in the context of significant caloric restriction likely augmented renal sensitivity and worsened hypothyroidism-mediated renal injury. Additionally, use of creatine might have resulted in a pseudoelevation of serum creatinine and an apparent underestimation of the effect of hypothyroidism on kidney function [5].

The significantly elevated titers of anti-thyroid peroxidase and anti-thyroglobulin antibodies supported the diagnosis of Hashimoto's thyroiditis as the cause of hypothyroidism in this case. Autoimmune hypothyroidism is often associated with other autoimmune disorders, such as type 1 diabetes mellitus and celiac disease, which show an autoimmune polyglandular propensity [4]. The presence of a strong family history of thyroid disease and type 1 diabetes in this patient points to a genetic autoimmune background.

From a clinical perspective, this case highlights several diagnostic and therapeutic criteria. Hypothyroidism needs to be included in the differential diagnosis of unexplained AKI, especially if combined with high CK or myopathic symptoms. In young patients, even severe thyroid hormone deficiency has been described as the cause of the first manifestation of rhabdomyolysis. Adolescents who use performance-enhancing supplements are a particularly susceptible population, particularly when they experience suboptimal hydration and nutrition. Early identification of hypothyroidism and initiation of thyroid hormone replacement, along with vigorous fluid therapy, is very effective and can result in quick (and almost complete) renal recovery. Late diagnosis may lead to severe renal failure, disturbances in electrolyte balance, and potentially life-threatening cardiovascular issues such as pericardial effusion or arrhythmias [1,5].

The majority of previously reported cases have either described hypothyroidism-induced AKI without rhabdomyolysis, rhabdomyolysis with only mild or potential renal involvement, or have been in middle-aged to older adults rather than adolescents. The uniqueness of our case is the simultaneous interplay of severe autoimmune hypothyroidism, rhabdomyolysis, dehydration, restrictive dieting, and creatine supplementation, causing AKI in a teenager, which has been reported to occur very rarely in the pediatric population.

## Conclusions

This case illustrates how autoimmune hypothyroidism in adolescents can present with acute kidney injury and rhabdomyolysis, particularly when combined with dehydration, a restricted diet, and creatine supplementation. The final patient showed a rapid clinical and biochemical response after intravenous hydration and thyroid hormone replacement, supporting the significant reversibility of renal failure with muscular dysfunction related to hypothyroidism when diagnosed early. This report, for the first time, highlights that a high index of suspicion for thyroid disease should be maintained in cases of unexplained AKI with high creatine kinase, particularly among young adults using performance-enhancing supplements. Early diagnosis, withdrawal of nephrotoxic or deceitful supplements, appropriate hydration, and early levothyroxine treatment are essential to avoid severe renal as well as systemic manifestations.
